# SIMcheck: a Toolbox for Successful Super-resolution Structured Illumination Microscopy

**DOI:** 10.1038/srep15915

**Published:** 2015-11-03

**Authors:** Graeme Ball, Justin Demmerle, Rainer Kaufmann, Ilan Davis, Ian M. Dobbie, Lothar Schermelleh

**Affiliations:** 1 Department of Biochemistry, University of Oxford, Oxford, UK; 2 Division of Structural Biology, Wellcome Trust Centre for Human Genetics University of Oxford, Oxford, UK

## Abstract

Three-dimensional structured illumination microscopy (3D-SIM) is a versatile and accessible method for super-resolution fluorescence imaging, but generating high-quality data is challenging, particularly for non-specialist users. We present *SIMcheck*, a suite of ImageJ plugins enabling users to identify and avoid common problems with 3D-SIM data, and assess resolution and data quality through objective control parameters. Additionally, *SIMcheck* provides advanced calibration tools and utilities for common image processing tasks. This open-source software is applicable to all commercial and custom platforms, and will promote routine application of super-resolution SIM imaging in cell biology.

The last decade has witnessed the spread of super-resolution light microscopy techniques that surpass the long-standing diffraction limit of optical resolution^1^,^2^,^3^. This has ushered in a second fluorescence microscopy revolution^4^, enabling researchers to better assess the location, structure, and dynamics of specifically labelled macro-molecules, thus beginning to bridge the gap between light microscopy and structural biology. Linear two- or three-dimensional structured illumination microscopy (SIM, 3D-SIM) provides multicolour optical sectioning with double the conventional resolution in the lateral^5^,^6^ and axial^7^,^8^ dimensions allowing fast 3-dimensional imaging of fixed and live specimens^9^,^10^,^11^,^12^. This is achieved by moving high-frequency information into the observable range of the microscope by frequency mixing with a fine-striped pattern of illumination. By imaging variations of the phase and angle of the illumination pattern this information can be computationally separated and recombined to reconstruct a super-resolution image with twice the frequency support in two or three dimensions^13^. Currently, the best practice in acquisition and processing of SIM data requires considerable expertise^14^,^15^ which represents a significant barrier to exploiting the full potential of SIM imaging^16^. Inaccurate instrument settings and suboptimal imaging conditions cause artifacts that are often difficult to distinguish from relevant structural features. Furthermore, there are no dedicated tools designed for thorough and unbiased quality assessment, and standardized post-processing of SIM data.

Here we describe *SIMcheck* (https://github.com/MicronOxford/SIMcheck and Supplementary Software), a user-friendly and simple solution to this challenge, which lowers the activation energy required for less experienced users and helps specialists and non-specialists to capture excellent quality 3D-SIM data and interpret it correctly. *SIMcheck* consists of a suite of plugins for the widely-used open-source application *ImageJ*^17^. It provides tools for assessment of the resolution, image quality and identification of sources of errors and artifacts in SIM imaging, as well as simplifying calibration and image handling steps. Additionally, *SIMcheck*’s output offers suggestions for how to address common deficiencies in SIM data. Raw and reconstructed data from any commercial SIM platform can be analysed with several options, and produce a variety of quality check outputs, a log text containing important metrics, and an output table with assessment of the data quality based on key statistics (Table 1, Supplementary Fig. S1). The names, function, type of output, areas of concern, and corresponding figures for each check are listed in Table 1. Corresponding log entries including interpretation guidelines are listed in Supplementary Table S2. A schematic representation summarizing how each check applies to the SIM imaging workflow is shown in Fig. 1, and the various functionalities of *SIMcheck* are colour-coded to correspond to the remaining figures.

## Structured illumination data checks

**Figure 1:**
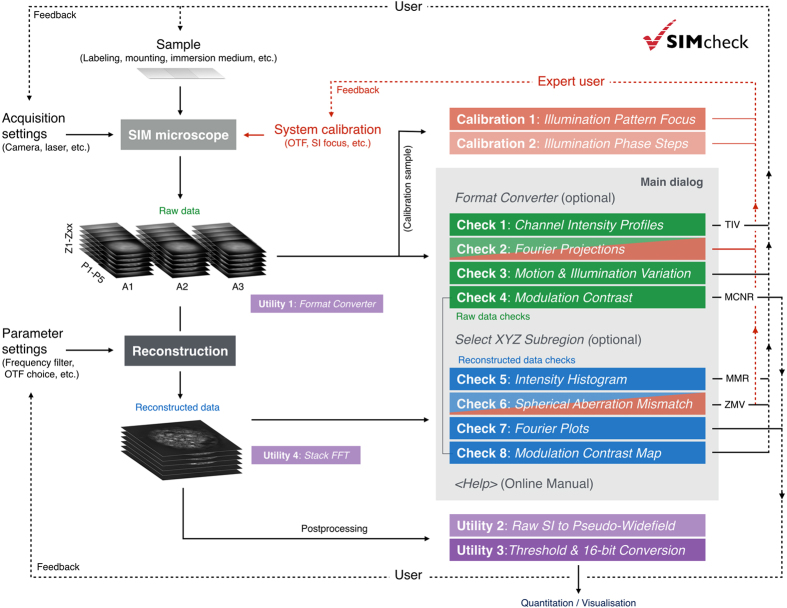
Integration of SIMcheck functionalities to the SIM imaging workflow. Solid lines represent the user’s workflow, and point from the various workflow steps to the applicable functions of *SIMcheck.* Dashed lines represent where SIMcheck’s output can inform user decisions, either in sample preparation, acquisition settings, reconstruction parameter settings, or system calibration. Colour codes are as follows: blue – raw data checks; green – reconstructed data checks; purple – utilities; red – applicable to expert users only.

**Figure 2:**
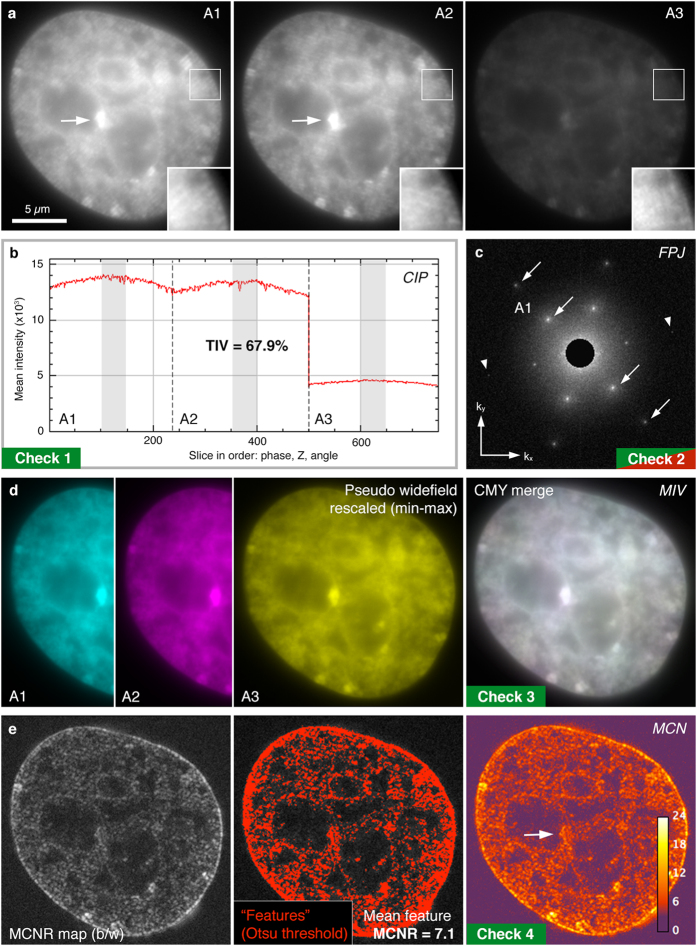
SIMcheck output for raw SIM data. (**a**) Representative images from a 3D-SIM dataset taken from a DAPI stained mouse C127 cell nucleus used for panels (**b**–**e**) (Supplementary Data S1). Data acquired on a GE OMX V3 Blaze instrument. Panels A1-A3 show the same plane with each of the three illumination angles. Insets show two-fold magnified and intensity-normalized view of the boxed region. Note the low contrast of the stripe pattern due to extensive out-of-focus blur contribution. (**b**) *Channel Intensity Profiles*, with total intensity variation (TIV) expressed as % within slices of a central 9-z-window in the three angles (marked light grey). In this example images from angle 3 show a markedly decreased intensity level (while there is little bleaching and intensity fluctuations) accounting for a total intensity variation of ~ 68%. (**c**) *Raw Fourier Projection* of the raw data in reciprocal space, with points of high-frequency information from first (inner) and second (outer spots) order stripes indicated by arrows. The presence of well-defined 1^st^ and 2^nd^ order spots in all three angles are a hallmark of optimal system calibration and generation of the structured illumination pattern. Arrowheads indicate less intense 2^nd^ order spots in angle 3. (**d**) *Motion & Illumination Variation* assembly of phase-averaged and intensity-normalized images for each angle (left three panels, pseudo-coloured in cyan, magenta and yellow). The grey-white appearance of the CMY-merged output image (right panel) indicates motion stability and evenness of the illumination. (**e**) *Modulation Contrast* output with grey values indicating the modulation contrast-to-noise ratio (MCNR) values (left panel, greyscale). These values are then Otsu thresholded to select features and calculate a mean feature MCNR (central panel). The final representation uses a custom look-up-table to generate a heatmap of local MCNR values (right panel). Arrows indicate regions of saturated pixels with accordingly lower modulation contrast.

Raw SIM data (Fig. 2a), prior to reconstruction, can be used to diagnose problems from three sources: (1) system calibration; (2) acquisition practices; (3) sample preparation (Table 1, Fig. 1). Deficiencies in any of these areas can lead to substantial artifacts after reconstruction, compromising the resolution of the images and making misinterpretation of results more likely. Loss or strong variations of signal intensity and the associated drop in signal-to-noise-ratio (SNR) in the raw data during acquisition can have a profound effect on the reconstruction output. Thus, the first raw data check is a *Channel Intensity Profile* (Fig. 2b) providing a plot of the average intensity for each z-plane, visualizing the rate of bleaching, intensity fluctuations, and differences in intensity between illumination pattern angles. Summary statistics report numerical values for each, together with the combined total intensity variation (TIV) over a central window of 9 z-planes ( = 135 images) (Supplementary Fig. S3).

To assess data quality in frequency space, the second check is a *Raw Fourier Projection* (Fig. 2c, Supplementary Fig. S4) of the entire z-stack. For samples that are sufficiently labelled and well contrasted, this allows the illumination pattern to be checked for clean first and second order spots, which represent high-frequency information (Fig. 2c, arrows). Raw data can also be viewed in reciprocal space for each individual image with the *Stack FFT* utility (Table 1, Supplementary Fig. S5).

The *Motion & Illumination Variation* check averages and intensity-normalizes the five phase-shifted images acquired for each plane for the different illumination pattern angles. It then assigns a false colour (cyan, magenta, yellow) to each angle (Fig. 2d). Subsequent merging of the angle information into one CMY image reveals intensity differences between temporally separated acquisitions as coloured sub-regions, whereas homogeneous data appears grey-white. This check can highlight uneven field-illumination or movement during acquisition, both of which can cause local reconstruction artifacts (Supplementary Fig. S5).

Intensity of the modulation contrast (or stripes) in the raw image is a key determinant of reconstructed image quality, as it critically affects the amount of frequency-shifted information that can be reassigned in the reconstruction process. Thus, the fourth check, *Raw Modulation Contrast* (Fig. 2e), calculates the ratio of the modulation contrast-to-noise (MCN) within the image by a specially devised Fourier analysis (Supplementary Fig. S6) and translates this to a heat map using a custom look-up-table (LUT). The check then reports an average modulation contrast-to-noise ratio (MCNR) for auto-segmented features and provides a recommended value for the high-frequency (Wiener) filter to be used for reconstruction (see **Methods**).

**Figure 3:**
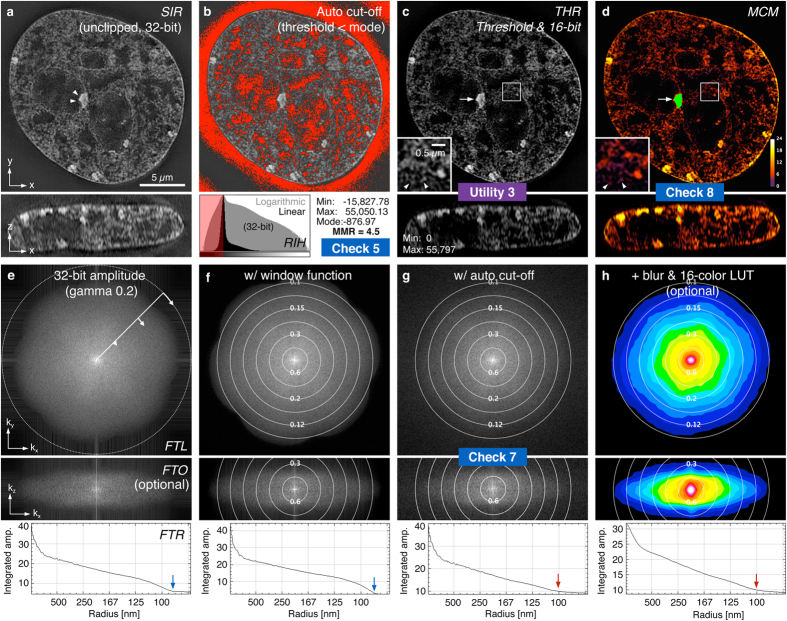
SIMcheck output for reconstructed SIM data. (**a**) Lateral and orthogonal cross section of reconstructed data used for panels (**b**–**h**), generated from raw data displayed in Fig. 2. (**b**) *Reconstructed Intensity Histogram* (bottom panel) showing the distribution of pixel intensities within the 32-bit data set on linear and logarithmic scales. Upper panel shows the areas below the mode intensity value that are discarded during thresholding. (**c**) The *Thresholding & 16-bit conversion* utility generates auto-thresholded composite TIFF stacks for further analysis and visualization (see also Supplementary Fig. S7). (**d**) *Modulation Contrast Map* combining the pixel intensity information of the reconstructed image with the colour information of respective MCN values (from Fig. 2e). Green colour indicates saturated pixels in the raw data that causes local reconstruction artifacts (arrow). Arrowheads in inset denote features in the nuclear interior of low modulation contrast (purple colour) in the corresponding raw data, compared to features with high underlying modulation contrast (orange-yellow) in the nuclear periphery. (**e**–**h**) Fourier spectra display variations of lateral FFT (FTL, top), orthogonal FFT (FTO, middle) and radial profile plot (FTR, bottom; orientation indicated in e, top panel). (**e**) 32-bit gamma 0.2 corrected amplitude Fourier spectrum of unclipped reconstructed data from (**a**). (**f**) Output with a window function applied to remove edge artifacts visible as horizontal and vertical stripes in (**e**). Overlaid concentric rings denote the respective spatial resolution (in μm). Note the distinct “flower” pattern with a prominent drop-off of the corresponding radial profile. The inflection point at ~90 nm (blue arrows) provides an approximation of the channel-specific frequency support. (**g**) Output of the mode-thresholded data shown in (**c**). The less pronounced frequency drop-off reflects the frequency mix of features and noise in the remaining (positive) intensity range after clipping the lower (negative) half of background (noise) intensities. The inflection point of the radial profile levelling with the amplitude background at ~100 nm (red arrows), provides an approximation of the effective resolution limit of features in the reconstructed data (see also Supplementary Fig. S8). (**h**) Output with optional additional colour-coding (right) applied in lateral and orthogonal Fourier spectra.

Analysis of reconstructed SIM data (Fig. 3) can diagnose problems that arise from system calibration errors, inappropriate acquisition settings, and from sub-optimal reconstruction parameters. The *Reconstructed Intensity Histogram* check (Fig. 3a,b) illustrates the proportion of pixel intensities above and below the mode (peak of the full-range histogram of the unprocessed image stack), and reports a minimum-to-maximum ratio (MMR) as a metric for the level of the feature intensity relative to reconstructed noise and intensity dips generated in the reconstruction process (Supplementary Fig. S7). Various factors can contribute to a low MMR, the most common being poor signal-to-noise and/or modulation contrast ratios in the raw data (e.g. by insufficient sample labelling, acquisition settings, or spherical aberration). Thus, the MMR serves as valuable indicator of the quality of reconstruction that can be used to diagnose various sources of error. Since the intensity values below the mode do not contain any feature information it is used as the default threshold for the *Threshold and 16-bit Conversion* utility (see below, Fig. 3c, Supplementary Fig. S7) and as an optional pre-processing step in the Fourier space analysis (below).

The reconstruction quality of specific structural features can vary in different regions of an image, and is dependent on variations in the underlying modulation contrast (e.g. by the local contribution of out-of-focus blur). To highlight these local variations, the *Modulation Contrast Map* check utilizes the MCN values from the *Raw Modulation Contrast* output to combine the colour look-up-table encoding the raw MCN values with the intensity information from the reconstructed data (Fig. 3d, Supplementary Fig. S9). Visual inspection allows assessment of how well the local modulation contrast in the raw data supports features observed in the reconstruction, with a very low level of underlying MCNR increasing the likelihood that an observed “feature” originates from reconstructed noise.

As with raw data, viewing reconstructed data in reciprocal space offers complementary information on image quality and resolution^18^. The *Reconstructed Fourier Plots* check contains three representations of the reconstructed data’s Fourier transform, in the lateral and axial directions, and a radial profile plot of the central slice in the lateral Fourier transform (Fig. 3e–h, top middle and bottom panels, Supplementary Fig. S8), respectively. The effective resolution of the reconstruction may be estimated, and errors resulting in atypical patterns can be identified (Supplementary Fig. S9). The axial Fourier plot is generated from an orthogonal cross section of the reconstructed data (Fig. 3a), to determine the frequency support/resolution of a 3D reconstruction along the z-axis (Fig. 3e–h, Supplementary Fig. S8).

**Figure 4:**
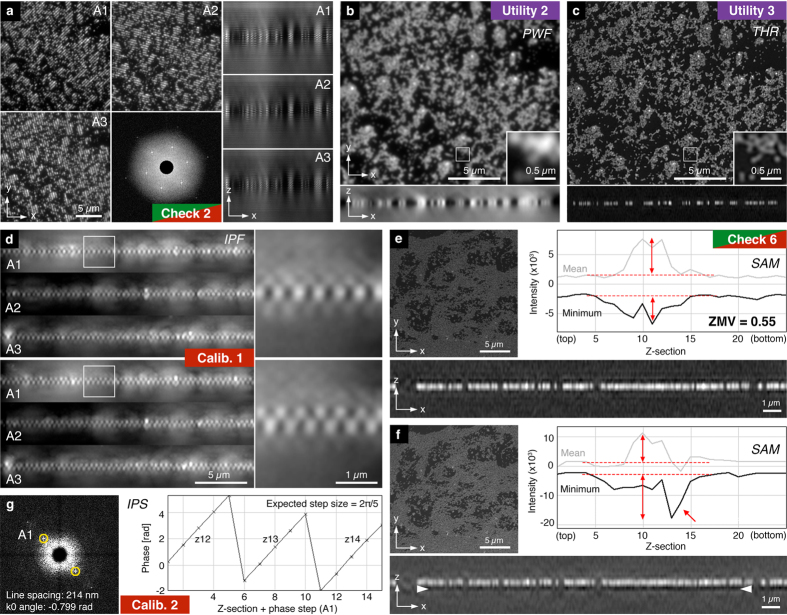
SIMcheck output for SIM calibration data and utilities. (**a**–**c**) 3D SIM data of a field of 0.1 μm diameter red fluorescent beads. (**a**) Representative central images of the raw data stack with the corresponding orthogonal views for each angle, and the *Raw Fourier Projection* output (bottom central panel). (**b**) Output from the *Raw SI to Pseudo-Widefield* utility, with orthogonal view and inset showing the dataset at conventional resolution. (**c**) Corresponding output from the *Threshold & 16-bit Conversion* utility demonstrating the increase in resolution and efficient rejection of out-of-focus blur. (**d**) *Illumination Pattern Focus* calibration tool applied to a field of red fluorescent beads imaged with two different system calibration settings showing orthogonal projections along the direction of the stripes for each angle. Top panel: single layer appearance with only weak, symmetric side lobes indicate good alignment of the axial illumination modulation with the focal plane. Bottom panel: zipper-like appearance indicates defocussing of the z-modulation for all three angles. (**e**,**f**) *Spherical Aberration Mismatch* check applied to a reconstructed dataset from a green fluorescent bead layer acquired under optimal (**e**) and suboptimal (**f**) imaging conditions, respectively. The intensity plot and the orthogonal cross section in **f** show a prominent dip in intensity underneath the bead layer (red arrow; white arrowheads), indicating a mismatch between sample/system conditions and the OTF used for the reconstruction. The corresponding z-minimum variation (ZMV) value relative to the average feature intensity (double arrows) is about three fold higher. (**g**) Output of the *Illumination Phase Step* utility of a dataset acquired from a green bead layer. The left panel shows a representative 2D FFT with the central area with the highest amplitude blocked. Yellow rings indicate auto-detected pixel positions of the highest intensity spots, normally associated with the first order stripes of the illumination pattern. The right panel displays a plot of the phase values in radians at these spots for all phase positions within a defined z-range (in this example ±1 z-sections around plane of best focus).

Lastly, the *Spherical Aberration Mismatch* check plots the minimum values for each z-section in comparison to the mean feature intensity and reports the z-minimum variation (ZMV) as a metric for the “depth of intensity dips” relative to the average feature intensity (Fig. 4e–f). Strong intensity dips and prominent side lobes around brighter features (“echo signals”), as well as reduced overall intensity are a hallmark of mismatch between the real point spread conditions in the sample and the ideal system behaviour encoded in the optical transfer function (OTF), the Fourier transform of the point spread function that is used for the reconstruction. In practice, this artifact type is mostly caused by increased levels of spherical aberration in the sample through variations in depth, refractive indices, temperature, etc. In addition, shifting of the illumination pattern focus (see below) can have a similar effect, which may call for a recalibration of the system. The ZMV metric is most useful as a relative measure to compare the same or very similar samples under variable acquisition conditions or reconstruction settings (e.g. to compare refractive index variation or different OTFs). These eight checks compose the core of *SIMcheck*’s functionality for the routine user, and are recommended to validate data for each sample type in an imaging experiment (Fig. 1).

### System calibration tools and image processing utilities

Additionally, *SIMcheck* contains two advanced system calibration tools, for use by imaging professionals to document system alignment, confirm system performance, and facilitate appropriate maintenance. Both tools require raw data containing an evenly covered field of sub-resolution fluorescent beads, or similar (Fig. 4a). The first of these checks is the *Illumination Pattern Focus*, which generates an orthogonal view of the illumination pattern for each illumination angle from the raw data (Fig. 4d, Supplementary Fig. S10). This can inform service of the instrument to ensure the highest intensity of modulation contrast at the image plane (Table 1). The second check analyses the *Illumination Phase Steps*, and reports on the reproducibility, and standard deviation of phase stepping for each illumination angle (Fig. 4g, Supplementary Fig. S10). This information allows for improved diagnostics, and can be an essential alignment tool for home-built or substantially modified instruments (Table 1).

Finally, *SIMcheck* contains a set of utilities to facilitate image handling and processing. The *Format Converter* takes data in .nd (Nikon) and .czi (Zeiss) file formats and reorders them into the default (GE OMX) format suitable for analysis with *SIMcheck* (Supplementary Fig. 3). *Raw SI to Pseudo-Widefield* averages images from the raw structured illumination data and assembles an image equivalent to conventional widefield illumination, allowing for comparison of super-resolution and conventional images (Fig. 4b, Supplementary Fig. S7). This can highlight the resolution improvement gained from using SIM, and identify artifactual bright features in regions with very low intensity in the pseudo-widefield image. As most downstream image processing and analysis tools require 16-bit images, inconsistency can arise in conversion of 32-bit reconstructed data into 16-bit formats. The *Threshold and 16-bit Conversion* utility discards intensities below an adjustable threshold (by default the stack’s modal intensity value), and fills the 16-bit range of the converted image (Figs 3c and 4c, Supplementary Fig. S7). This tool facilitates higher standards in data handling and reporting for image analysis.

## Conclusion

Understanding how to verify instrument performance, tune parameters, and recognize artifacts are essential to obtaining optimal and reproducible super-resolution SIM data. The tools contained in *SIMcheck* enable general users and specialists to diagnose problems they encounter in acquiring and processing SIM data. In combination with careful system calibration and sample preparation protocols, as well as a comprehensive approach to experimental design, execution, and analysis, *SIMcheck* helps SIM systems perform to their full capability. This toolbox will facilitate establishing best practice in generating and interpreting SIM images, making the technique much more accessible to the wider biology community, and ensuring super-resolution imaging fulfils its great potential.

## Methods

### Installation and quick start

The *SIMcheck* code is designed to run with the standard *ImageJ* (1.49 or newer) software package, requiring in addition a current version of *Bioformats Importer* plugin installed. Alternatively, it can be run with an up-to-date *Fiji* distribution of *ImageJ*^19^ that includes the plugin by default. For installation the SIMcheck.jar file is copied into the Plugin folder of *ImageJ/Fiji* before restarting. Installing with *Fiji* offers the possibility for automatic updates by activating the *SIMcheck* update site in the *ImageJ Updater* accessible from the top down menu (*Help -> Update Fiji -> Manage update sites*). When importing raw data through *Bioformats Importer*, the “Stack Viewing” option must be set to “Hyperstack”. The suite with four *Raw Data Checks* and four *Reconstructed Data Checks* can either be run in batch mode selected from the main *Run SIMcheck* dialog, or as stand-alone tests with extended options (Supplementary Fig. S1). In addition, two *System Calibration Tools* and four *Image Processing Utilities* are provided. The main dialog prompts the user to define the raw and/or reconstructed dataset to be analysed, the data format (GE OMX (default), Zeiss Elyra or Nikon N-SIM for conversion) and the camera bit-depth of the imaging system (to identify saturated pixels). It further enables cropping of both raw and reconstructed datasets to the same boundaries in x, y and z for specific analysis of defined sub-regions. The <help> button in the main dialogue links to an *Online Manual* that provides step-by-step guidelines how to use each tool and interpret its outcome. Note that the *Reconstructed Intensity Histogram (RIH)* and *Spherical Aberration Mismatch (SAM)* checks (see below) rely on reconstructed data still comprising the full histogram range. Clipping the low intensity range (the default setting in commercial reconstruction software) will also affect the output of the *Reconstructed Fourier Plots* (see below).

### Raw Data Checks

#### Channel Intensity Profile (CIP)

The check plots the absolute (slider position 1) and relative (slider position 2) average intensity for each plane (in the order: phase, z, angle, time) where each channel is assigned an arbitrary colour: 1st channel = red, 2nd = green, 3rd = blue, subsequent channels = black (Supplementary Fig. S3). Several intensity statistics are also calculated, including a summary statistic, total intensity variation (TIV) reporting the min-max range as a percentage of the maximum intensity for a 9-z-window about the central slice, over all phases and angles used in the reconstruction. A window size of ± 4 z-sections was chosen, as the algorithm requires adjacent sections of the raw data stack to reconstruct a single 3D-SIM section and thus could contribute to potential artifacts. Three more specific statistics based on mean slice intensities help identify the type of intensity fluctuations present: (1) estimated intensity decay, which denotes the relative fluorescence decay per angle (averaged over all angles), as a measure of photobleaching; (2) maximum intensity difference between angles, which denotes the maximum difference between any two angles as a percentage of the maximum angle intensity; (3) relative intensity fluctuations (“flicker”), which denotes the average over all phases and angles of the min-max range over a 9-z-window about the central slice.

#### Raw Fourier Projection (FPJ)

This check applies a Gaussian windowing of 6% of the image width prior to carrying out a 2D Fourier transform (FFT) calling ImageJ’s *FHT* routine, which returns a 32-bit logarithmically scaled amplitude squared [log(amplitude^2^)] Fourier spectrum that particularly amplifies the relevant high frequency range. Subsequently a maximum intensity projection over all phases and angles is generated from the resulting FFT stacks for each channel. The central circular region (diameter 1/8 of image width) is then filled with the minimum amplitude value before rescaling (min-max) to provide optimal contrast (Supplementary Fig. S4). The resulting Fourier projection highlights spots corresponding to the first and second order stripe frequencies in the illumination pattern for each angle. This check requires that the sample fills a significant proportion of the volume and that the signal-to-noise is adequate, in order to see second order spots. Sparsely fluorescent samples, such as isolated point-like objects would not show prominent spots even though the data quality may be sufficient. Also, FFT of 2D-SIM images acquired in TIRF mode would not show any spots, because the detection numerical aperture (NA) of the TIRF objective is significantly smaller than the excitation NA, preventing the detection of high frequency stripes in the excitation. Because of these specific requirements the check is by default not activated when running *SIMcheck* from the main dialog.

#### Motion & Illumination Variation (MIV)

The phases (default 5) from each angle at each z-plane are averaged and then each angle (assumes 3) is intensity-normalized before assigning a false colour cyan, magenta, or yellow (Supplementary Fig. S5). In this way features present in all angles will appear grey to white, while either movement of certain features or variations in illumination from the different angles, will exhibit an angle-specific colour. The colour scheme chosen here is intended to make the distinction between angles and channels clear. The reconstruction algorithm assumes that all features are sampled at each angle, and features that move significantly during the acquisition of a 3D frame or experience very different illumination intensity for different angles will result in artifacts. Of note, because of the linear polarization of excitation along the direction of stripes, some regional variations would also be observed in case of samples with polarization-dependent fluorescence excitability, such as actin fibres or microtubules, without causing any artifacts.

#### Modulation Contrast-to-Noise (MCN)

To obtain a measure of the local stripe contrast, the following calculation is carried out for each voxel in the real 3D image: (1) A variance stabilizing Anscombe transform^20^ is performed so that noise follows an approximately Gaussian, rather than Poissonian, distribution. (2) A z-window is selected as 2*z + 1 (z = number of z-planes above and below to be combine with each z-plane) and all raw phase images within this window are stacked (the default z-window of ±1 z-sections increases signal-to-noise to a similar extent to the “band filtering” performed during reconstruction). These phase series are Fourier-transformed using a hand-coded multithreaded 1D discrete Fourier transformation along the dimension of the different phases. The result of this 1D Fourier-transformation allows separating the different frequency components of the raw data (Supplementary Fig. S6). (3) The power of the frequency components corresponding to the illumination pattern modulation are divided by the standard deviation of the highest frequency component for the same z-plane (taken to be dominated by noise). The frequency components of first and second order in the Fourier transformed stack are located at plane numbers: *L*_FT_ * *O*/*N*_p_ + 1, with L_FT_ = length of the above Fourier transformed data stack (e.g. 5 phases * 3 z-sections = 15), *N*_p_ = number of phase shifts during data acquisition, and *O* = order number (1 or 2). The modulation-contrast-to-noise-ratio value is calculated as 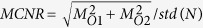. Average modulation contrast for each channel is estimated using *ImageJ*’s Otsu auto-thresholding routine with the stack histogram. Finally, a suggested Wiener filter parameter is given (for GE Healthcare’s SoftWoRx SI reconstruction) based on the following empirical formula: Wiener filter constant, w = 0.17/MCNR^2^.

### Reconstructed Data Checks

#### Reconstructed Intensity Histogram (RIH)

Linear- and logarithmic-scaled intensity histograms (black and grey, respectively) showing relative contribution of values below the modal intensity value for background regions, which are due to reconstructed noise and ringing artifacts at the edge of high-intensity features (Supplementary Fig. S7). Only intensities at the extremes of the histogram are considered: by default the lowest and highest 0.05% of intensities, subject to a minimum of 100 pixels. The min-to-max ratio (MMR) reported is calculated as: Max* − Mode/|Min* − Mode|; where Min* and Max* are the averages of the lowest and highest intensity pixel values, respectively. The number of pixels in each average is also shown in case artifacts such as zeros from truncation in processing or saturated pixels in data collection are significantly skewing the values. For meaningful results, the check requires untrimmed reconstructed data covering the full intensity range, such that in the reconstruction settings of commercial software the option “discard negatives” (GE), “baseline cut mode” (Zeiss) or equivalent must be deactivated.

#### Spherical Aberration Mismatch (SAM)

This check plots the minimum values and the mean feature intensity values (selected by Otsu auto-thresholding) in each slice. The summary statistics reports the z-minimum variation (ZMV) as the standard deviation of the slice minima, normalized by the average feature intensity (Fig. 4e,f). The ZMV metric is useful as a relative measure for artifacts caused by spherical aberration mismatch between the sample and the optical transfer function (OTF) when comparing datasets of similar characteristics, but acquired under different imaging conditions or reconstructed with different OTFs. This check requires untrimmed data, and it works best with flat, high-contrast samples (e.g. layers of beads) and is by default not activated when running *SIMcheck* from the main dialog.

#### Reconstructed Fourier Plots (FTL, FTO, FTR)

This check carries out a 2D fast Fourier transform (FFT) for each slice of the reconstructed data set to return a 32-bit amplitude Fourier spectrum with a gamma correction applied (gamma = 0.2) to enhance visibility in the higher frequency ranges. It outputs a stack of transformed xy-slices (Fourier Transform Lateral, FTL) displaying absolute amplitude levels with a pre-set scaling (between 2 and 40), overlaid with concentric rings that indicate the corresponding spatial resolution (in μm), and a radially integrated profile plot of the central z-slice in the FTL result (Fourier Transform Radial, FTR) (Fig. 3e–h). The data is by default pre-processed by subtracting the lower half of the background noise intensities, defined by the stack mode (i.e. the peak of the stack histogram), to reduce the frequency contribution of reconstructed noise in the resulting FFT and to allow comparability with datasets that have already been clipped accordingly by the reconstruction software. It is important to note that automatic clipping only provides meaningful results if the data contains a sufficient fraction of label-free background, so that only pixels within background regions are set to zero. If the field of view (or cropped region of interest) contains very little background, the modal value may provide an erroneously high cut-off, which can lead to artificial “sharpening” of structures, with the radial profile extending beyond the theoretical frequency support (no inflection point). In this case the cut-off limit should be set manually (for 32-bit data acquired and reconstructed with the correct camera/background offsets, this value is typically around the zero level).

The stand-alone plugin allows additional options and combinations thereof: (1) deactivate auto-cut-off, or apply a manual cut-off value (default = 0); (2) apply a window function (6% width) to remove horizontal and/or vertical stripe artifacts in the Fourier spectrum (e.g. if no cut-off is applied or if sample features extend over the image boundaries); (3) return auto-scaled (min-max) gamma 0.2 corrected amplitude Fourier spectrum, or a log(amplitude^2^) power spectrum with subsequent mode-max rescaling of each slice to provide a normalized FFT stack with further enhanced higher frequency range contrast over the default display option; (4) additional blurring and false-colouring with a 16-colour look-up-table (LUT) to highlight frequency dependent gradient characteristics of the amplitudes in the FFT plots; (5) display an axial FFT plot from the central re-sliced xz-section interpolated to generate isotropic voxels (Fourier Transform Orthogonal, FTO) (Supplementary Fig. S8). Note that re-slicing is carried out using *ImageJ*’s built-in command, and the radial profile plot is generated using a modified version of Paul Baggethun’s plugin: http://rsb.info.nih.gov/ij/plugins/radial-profile.html. Overlaid rings can be removed or flattened down using the respective commands from the *ImageJ/Fiji* the top down menu (*Image -> Overlay -> Remove Overlay*, or *Flatten*).

#### Modulation Contrast Map (MCM)

The *Modulation Contrast Map* displays the reconstructed data with the colour information from the raw data MCN plugin multiplied with the intensity for each pixel, normalized to the maximum intensity in the image. The result is an RGB image where the mapped colour of reconstructed features (real or artificial) indicates the underlying modulation contrast in the corresponding raw data. Lastly, voxels that are saturated in one or more angles in the raw data (based on camera bit depth) are coloured green to highlight regions with missing stripe information that may cause local artifacts (Fig. 3d, Supplementary Fig. S9)

### System Calibration Tools

#### Illumination Pattern Focus (IPF)

This is a calibration check designed to work best with a dense single layer of sub-resolution size fluorescent beads that provide a high-contrast images of continuous stripes. Data are pre-processed to normalize intensity and correct slice-to-slice flicker using a simple ratio of mean slice intensities (Supplementary Fig. S10). The first phase for each angle is taken and rotated so that it is vertically aligned. The rotated sub-stacks are re-sliced orthogonally for an xz-view, and a maximum-intensity projection is performed along the direction of the stripes. The separate angles are displayed and stacked as a montage providing a visual representation of the axial illumination pattern where the pattern’s alignment with the image plane in focus can be assessed.

#### Illumination Phase Steps (IPS)

This calibration check again requires a dense sample such as a bead lawn. Raw data slices are Fourier-transformed, the high intensity regions around the origin and along both horizontal and vertical axes, are masked, and first order spots are detected over a specified z-slice range using *ImageJ*’s built-in *Process > Find Maxima* command (Supplementary Fig. S10). The phase of the illumination pattern thus detected are plotted as a phase series (radians, unwrapped) for each step. Numerical statistics report on phase step accuracy (phase step standard deviation) and phase offset stability (phase offset standard deviation).

### Image Processing Utilities

#### Format Converter (GE OMX)

Converts Zeiss and Nikon format SIM data into the GE OMX ’s “CPZAT” (channel, phase, z-slice, angle, time) dimension ordering, since all of the check plugins assume and require this dimension ordering to work correctly (Supplementary Fig. S3).

#### Raw SI to Pseudo Widefield (PWF)

Generates a pseudo-widefield image by (1) averaging all phases and angles for each slice in a raw SIM dataset and (2) rescaling in x-y by a factor of two using a bicubic interpolation to adapt the pixel size to the reconstructed data for direct comparison (Fig. 4b, Supplementary Fig. S7). Optionally, “simple ratio” intensity normalization may be applied to correct for intensity fluctuations and bleaching.

#### Threshold and 16-bit Conversion (THR)

Discards intensities below a threshold (by default automatic to the stack mode, or manually-defined per channel) and converts the remainder to 16-bit data, filling the 16-bit range. The dataset may be saved as composite tiff-stack for further processing and analysis (Fig. 4c, Supplementary Fig. S7).

#### Stack FFT

Applies a 2D Fourier Transform to each slice in a stack. By default this uses an extension ImageJ’s FHT class to produce log-scaled amplitude images for the complex FFT result, and implements Gaussian windowing of 6% of the image width prior to padding with zeros and transformation (Supplementary Fig. S4). Optionally, the windowing percentage can be varied and the scaling method changed to a gamma correction of choice.

For further information and guidance on applying *SIMcheck* to your data, see the *SIMcheck* user manual at http://www.micron.ox.ac.uk/microngroup/software/SIMcheck.html

Source data from all commercial platforms is available for testing as Supplementary Data (https://www.dropbox.com/sh/4deho8b9cyjb8u7/AABWRZvfGu6M2B6hnmz_qLxxa?dl=0). Details on sample preparation and microscope system configuration are available upon request.

## Additional Information

**How to cite this article**: Ball, G. *et al.* SIMcheck: a Toolbox for Successful Super-resolution Structured Illumination Microscopy. *Sci. Rep.* **5**, 15915; doi: 10.1038/srep15915 (2015).

## Supplementary Material

Supplementary Information

## Figures and Tables

**Table 1:**
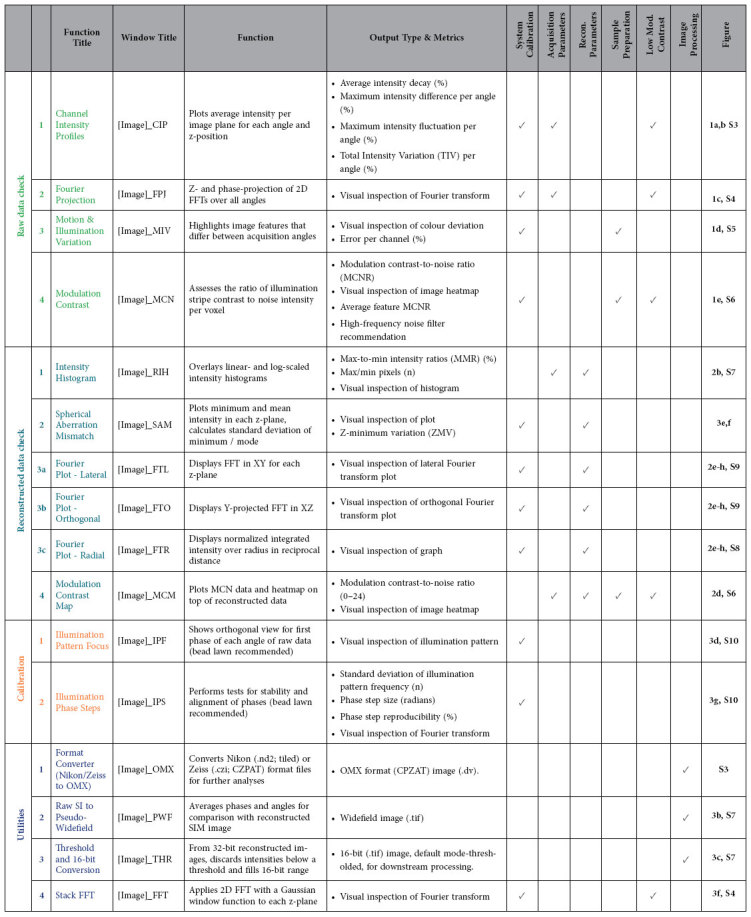
*SIMcheck* functionalities.

Each check is listed by: title; window title of the output; the main function of the check; and the expected output and metrics. The category to which each check or tool may apply is indicated by checkmarks. The corresponding Figures in the main text and Supplementary Information are listed, showing examples and containing more detailed instructions and explanations for each check respectively. Additional notes and interpretation guidelines are listed in Supplementary Table S2.
