# Erratum: SIMcheck: a Toolbox for Successful Super-resolution Structured Illumination Microscopy

**DOI:** 10.1038/srep20754

**Published:** 2016-02-05

**Authors:** Graeme Ball, Justin Demmerle, Rainer Kaufmann, Ilan Davis, Ian M. Dobbie, Lothar Schermelleh

Scientific Reports 5: Article number: 1591510.1038/srep15915; published online: 11032015; updated: 02052016

In the Supplementary Information file originally published with this Article, the details of the beta testers in Supplementary Table S11 were incorrect. These errors have been corrected in the Supplementary Information that now accompanies the Article.

