# Supplementary material for: SIMcheck: a Toolbox for Successful Super-resolution Structured Illumination Microscopy
Source: Sci Rep. 2015 Nov 3;5:15915. doi: 10.1038/srep15915 (PMC4648340; doi:10.1038/srep15915)
Supplement: Supplementary Information [file srep15915-s1.pdf]

Supplementary Information

SIMcheck: a Toolbox for Successful Super-resolution Structured Illumination Microscopy

Graeme Ball[1,3], Justin Demmerle[1], Rainer Kaufmann[1,2], Ilan Davis[1], Ian M. Dobbie[1] & Lothar Schermelleh[1]

[1]Department of Biochemistry, University of Oxford, Oxford, UK.

[2]Division of Structural Biology, Wellcome Trust Centre for Human Genetics University of Oxford, Oxford, UK.

[3]Current Address: Dundee Imaging Facility, School of Life Sciences, University of Dundee, Dundee, UK.

Correspondence should be addressed to L.S. (lothar.schermelleh@bioch.ox.ac.uk).

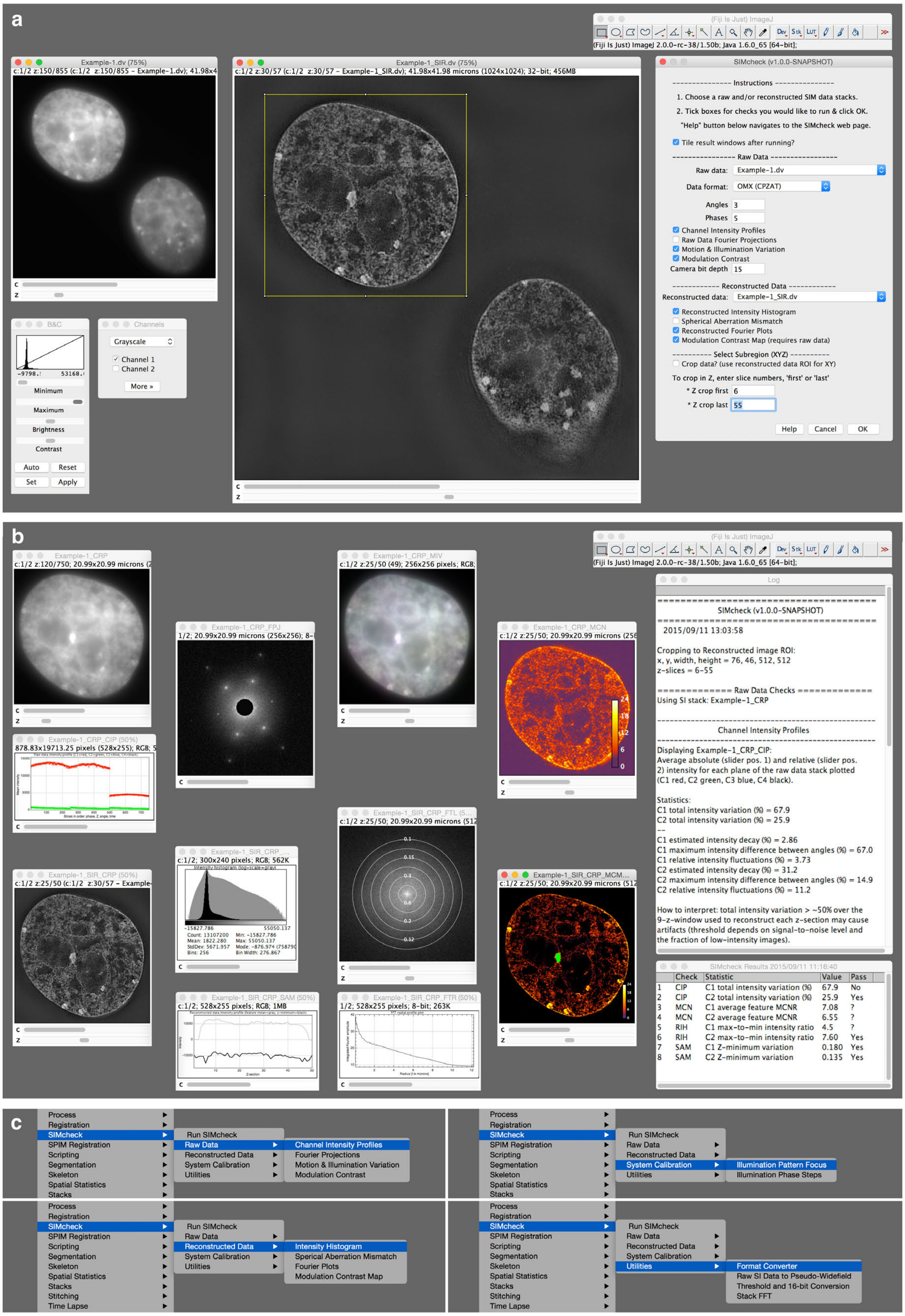

a
(Fiji Is Just) ImageJ 2.0.0-rc-38/1.50b; Java 1.6.0_65 [64-bit];
Example-1.dv (75%)
Example-1_SIR.dv (75%)
c:1/2 z:30/57 (c:1/2 z:30/57 - Example-1_SIR.dv); 41.98x41.98 microns (1024x1024); 32-bit; 456MB
B&C
Minimum
Maximum
Brightness
Contrast
Auto
Reset
Set
Apply
Channels
Grayscale
Channel 1
Channel 2
More »
SIMcheck (v1.0.0-SNAPSHOT)
Instructions
1. Choose a raw and/or reconstructed SIM data stacks.
2. Tick boxes for checks you would like to run & click OK.
"Help" button below navigates to the SIMcheck web page.
Tile result windows after running?
Raw Data
Raw data: Example-1.dv
Data format: OMX (CPZAT)
Angles 3
Phases 5
Channel Intensity Profiles
Raw Data Fourier Projections
Motion & Illumination Variation
Modulation Contrast
Camera bit depth 15
Reconstructed Data
Reconstructed data: Example-1_SIR.dv
Reconstructed Intensity Histogram
Spherical Aberration Mismatch
Reconstructed Fourier Plots
Modulation Contrast Map (requires raw data)
Select Subregion (XYZ)
Crop data? (use reconstructed data ROI for XY)
To crop in Z, enter slice numbers, 'first' or 'last'
* Z crop first 6
* Z crop last 55
Help
Cancel
OK
b
Example-1_CRP
Example-1_CRP_FPJ
Example-1_CRP_MIV
Example-1_CRP_MCN
Example-1_CRP_CIP (50%)
Example-1_SIR_CRP (50%)
Example-1_SIR_CRP_
Example-1_SIR_CRP_FTL
Example-1_SIR_CRP_MCM...
Example-1_SIR_CRP_SAM (50%)
Example-1_SIR_CRP_FTR (50%)
Log
SIMcheck (v1.0.0-SNAPSHOT)
2015/09/11 13:03:58
Cropping to Reconstructed image ROI:
x, y, width, height = 76, 46, 512, 512
z-slices = 6-55
Raw Data Checks
Using SI stack: Example-1_CRP
Channel Intensity Profiles
Displaying Example-1_CRP_CIP:
Average absolute (slider pos. 1) and relative (slider pos.
2) intensity for each plane of the raw data stack plotted
(C1 red, C2 green, C3 blue, C4 black).
Statistics:
C1 total intensity variation (%) = 67.9
C2 total intensity variation (%) = 25.9
C1 estimated intensity decay (%) = 2.86
C1 maximum intensity difference between angles (%) = 67.0
C1 relative intensity fluctuations (%) = 3.73
C2 estimated intensity decay (%) = 31.2
C2 maximum intensity difference between angles (%) = 14.9
C2 relative intensity fluctuations (%) = 11.2
How to interpret: total intensity variation > ~50% over the
9-z-window used to reconstruct each z-section may cause
artifacts (threshold depends on signal-to-noise level and
the fraction of low-intensity images).
SIMcheck Results 2015/09/11 11:16:40
Check Statistic Value Pass
1 CIP C1 total intensity variation (%) 67.9 No
2 CIP C2 total intensity variation (%) 25.9 Yes
3 MCN C1 average feature MCNR 7.08 ?
4 MCN C2 average feature MCNR 6.55 ?
5 RIH C1 max-to-min intensity ratio 4.5 ?
6 RIH C2 max-to-min intensity ratio 7.60 Yes
7 SAM C1 Z-minimum variation 0.180 Yes
8 SAM C2 Z-minimum variation 0.135 Yes
c
Process
Registration
SIMcheck
SPIM Registration
Scripting
Segmentation
Skeleton
Spatial Statistics
Stacks
Stitching
Time Lapse
Run SIMcheck
Raw Data
Reconstructed Data
System Calibration
Utilities
Channel Intensity Profiles
Fourier Projections
Motion & Illumination Variation
Modulation Contrast
Illumination Pattern Focus
Illumination Phase Steps
Intensity Histogram
Sperical Aberration Mismatch
Fourier Plots
Modulation Contrast Map
Format Converter
Raw SI Data to Pseudo-Widefield
Threshold and 16-bit Conversion
Stack FFT

Supplementary Figure S1 | *SIMcheck* screenshots. (**a**) Screenshot of the *SIMcheck* main dialog after being opened in *Fiji/ImageJ* (top right, default settings), with raw data of DAPI stained mouse C127 cell nuclei (left, **Supplementary Data 1a**) and the corresponding, unmodified, reconstructed data (centre, **Supplementary Data 1b**). The upper cell is highlighted for cropping (512x512 pixel ROI using *Edit* > *Selection* > *Specify…*), limiting *SIMcheck* analysis to the selected region. Useful *B&C* and *Channels* tools (bottom left) are opened automatically when running *SIMcheck* from the main dialog box. (**b**) Screenshot of *SIMcheck* output with all raw and reconstructed data checks for channel 1 (DAPI; channel 2 not shown). In addition, a *Log* window providing a short description, extended results and interpretation for each check (right centre), and a *SIMcheck Results* window summarizing the most important metrics (right bottom). (**c**) Four screenshots illustrating the menu structure for all stand-alone plugins.

Supplementary Table S2 | Notes and interpretation guidelines

		Function Title	Notes / About	How to interpret
Raw data check	1	Channel Intensity Profiles	Average absolute (slider pos. 1) and relative (slider pos. 2) intensity for each plane of the raw data stack plotted (C1 red, C2 green, C3 blue, C4 black).	Total intensity variation > ~50% over the 9-z-window used to reconstruct each z-section may cause artifacts (threshold depends on signal-to-noise level and the fraction of low-intensity images).
	2	Fourier Projection	Maximum intensity projection of log(amp^2) 2D FFT stack, with central area masked, and rescaled (min-max) to improve contrast of the relevant frequency range.	Look for clean 1st & 2nd order spots, similar across angles. Note that spot intensity depends on image content.
	3	Motion and Illumination Variation	Each angle phase-averaged, normalized, and false-colored (A1 cyan, A2 magenta, A3 yellow).	Non-white areas indicate differences between angles due to drift, floating particles or illumination variations.
	4	Modulation Contrast	Modulation contrast-to-noise ratio (MCNR) image. Average feature MCNR selected by auto-thresholding (Otsu).<br><br>Estimated Wiener filter parameter is valid for OMX data reconstruction (SoftWoRx) only.	Colour LUT indicates MCNR value:<br>• purple is inadequate (<4)<br>• red is low to moderate (4-8)<br>• orange is good (8-14)<br>• yellow-white is very good to excellent (>14)
Reconstructed data check	1	Intensity Histogram	Intensity counts in black (linear) & grey (log-scale).<br><br>MMR is calculated as the ratio of the averaged 0.001% highest (Max*) and lowest (Min*) intensity pixels in a 32-bit stack, centred at the stack mode (assumed to be the centre of the noise distribution), that is:<br>Max* - Mode / |Min* - Mode|	Max-to-min intensity ratio ranges: MMR <3 is inadequate, 3-6 is low, 6-12 is good, >12 excellent. For valid results, the data set must contain sufficient background areas (so that the mode reflects background) and should be constrained to z-slices containing features.<br><br>N.B. MMR statistic is only valid for unclipped data (reconstruction option 'discard negatives' or 'baseline cut mode' deactivated).
	2	Spherical Aberration Mismatch	Z-section minimum (black) and mean feature intensity (grey).<br><br>Z-minimum variation (ZMV) is calculated as the standard deviation of z-section minimum intensity normalized to the average feature intensity.	High ZMV indicates spherical aberration mismatch between the sample and the optical transfer function used for the reconstruction.<br>Typically this is seen as a dip in the minimum intensity plot at the sample boundary. Note that the absolute value depends on image content.<br>N.B. The ZMV statistic is only valid for unclipped data (reconstruction option 'discard negatives' or 'baseline cut mode' deactivated).
	3	Fourier Plots	Fourier Transform Lateral (XY; resolution rings in microns)<br>[Optional: Fourier Transform Radial profile (lateral, central Z)].<br><br>By default the reconstructed data are<br>(1) cropped to the mode intensity value and<br>(2) Fourier transformed and scaled by a gamma function (gamma=0.2).	Fourier plots highlight potential artifacts and indicate effective resolution:<br>• Spots in Fourier spectra indicate periodic patterns.<br>• A flattened Fourier spectrum (a plateau in the radial profile) indicates the lack of real high frequency signal and therefore poor resolution.<br>• Asymmetric Fourier spectra indicate angle-specific decreases in resolution due to any of the following: angle-to-angle intensity variations; angle-specific illumination pattern ('k0') fit error; or angle-specific z-modulation issues.
	4	Modulation Contrast Map	MCN: Reconstructed data color-coded according to the underlying Modulation Contrast-to-Noise Ratio (MCNR) in the raw data.	The MCNR map indicates local variations in reconstruction quality, e.g. variations in out-of-focus blur contributions due to feature density, or uneven patterning of the structured illumination.<br><br>MCNR values: 0-4 purple (inadequate), to 8 red (acceptable), to 12 orange (good), to 18 yellow (very good), to 24 white (excellent).<br><br>Saturated pixels detected in the raw data according to selected bit-depth are false-colored green.
Calibration	1	Illumination Pattern Focus	Projected side view along the illumination stripes (phase 1 only) for each angle to illustrate alignment of the illumination pattern's z-modulation with the focal plane. Slice intensities are normalized to correct for intensity variations.	100 nm bead layer in the focal plane should display distinct intensity modulation (side view of first order stripes. Intensity dips above and below the intensity peaks should be balanced. A "zipper-like" appearance (i.e. two staggered layers of modulated intensities) indicates defocussing of the z-modulation against the focal plane. All angles should have the same characteristics.

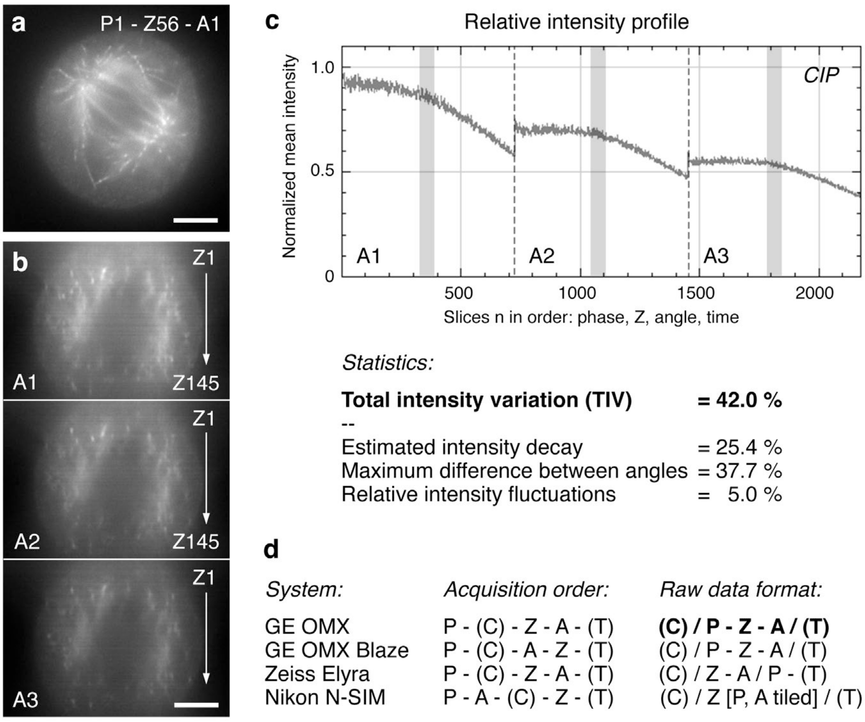

Supplementary Figure S3 | Generation of *Channel Intensity Profiles (CIP)*. (**a**) Raw data image stack of a HeLa cell immunostained for microtubules (detected with Alexa Fluor 488 conjugated secondary antibodies) acquired on an OMX V2 instrument (**Supplementary Data 2a**). From a central z-section (Z56), the image for phase step 1 (P1) of angle 1 (A1) is shown. (**b**) Orthogonal view of raw data stack with acquisition angles A1 - A3 and z-slices shown with the first slice (Z1) at the top, and the last slice (Z145) at the bottom for each angle (only P1 shown). Scale bar: 5 µm. (**c**) *SIMcheck* output with the relative mean intensity of each frame plotted on the y-axis, and the order of the slices, separated by angle, on the x-axis. Five phase steps (P1-5) are acquired per z-slice and angle, resulting in 145 x 5 = 725 images per angle in the dataset. The employed OMX V2 system uses a mechanical phase grating for the pattern generation and acquires image stacks for different rotation angles sequentially. Accordingly, fluorescence intensity decreases through the z-stack and with each consecutive angle due to photobleaching. Below is the corresponding *SIMcheck* log output, providing the total intensity variation (TIV) within a window of 9 central z-sections contributing to reconstruct one super-resolution z-section (marked light grey). In addition statistics on the average decay rate per angle, maximum intensity difference between angles and relative intensity fluctuations are provided to diagnose selective contributions to the TIV. (**d**) Table comparing the acquisition order and raw data format of the three commercially available SIM platforms. GE's OMX platform comes with either a rotary diffraction grating (V2/V3) where angle rotation is the last step, or a rapid angle-switching device (V3/V4 Blaze), which has a different acquisition order. It should be noted that acquisition on these instruments may be switched by the user between "all channel then Z" or "all Z then channel". While the raw data format varies between manufacturers, *SIMcheck* is written to accommodate by default the GE data format, and therefore datasets from other manufacturers need to be converted either from the main dialog or separately with the *Format Conversion* tool prior to analysis with any of the stand-alone checks and utilities.

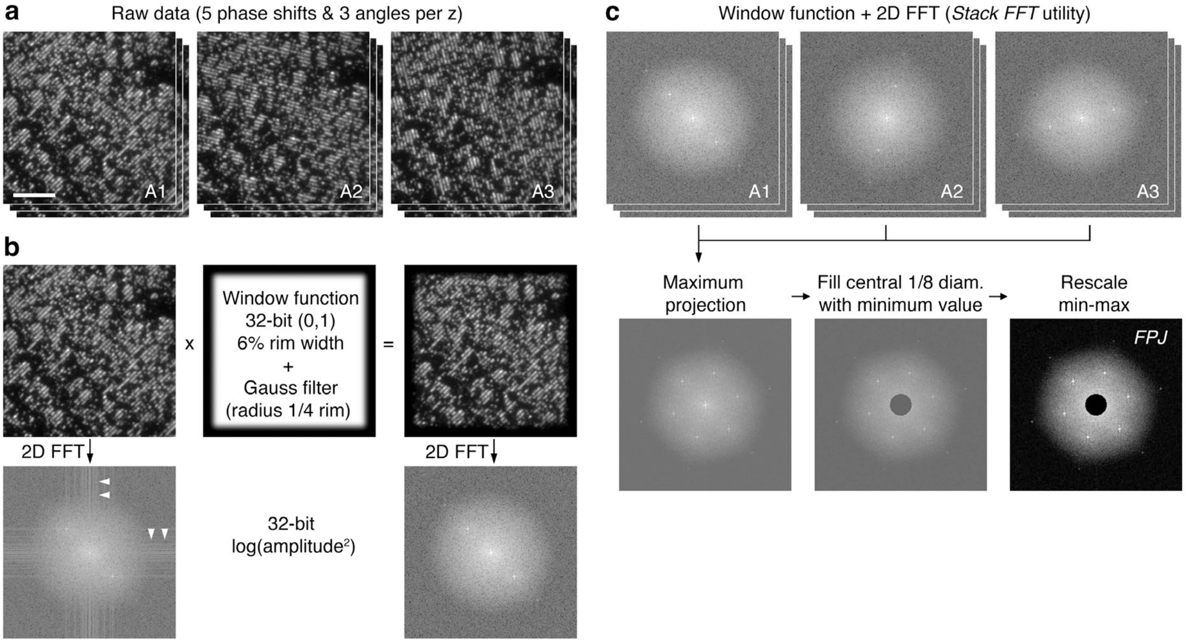

Supplementary Figure S4 | Generation of raw data *Fourier Projection (FPJ)*. (**a**) Raw data from a field of green 110 nm diameter fluorescent beads (FluoSphere 505/515, Life Technologies), demonstrating how each illumination angle is considered as a separate z-stack for the construction of the *Raw Fourier Projection (FPJ)* check. Data acquired on a GE OMX V3 Blaze instrument (**Supplementary Data 3a**). Scale bar: 5 µm. (**b, c**) Illustration of intermediate processing steps to generate the final *FPJ* output. (**b**) A 2D fast Fourier transform (FFT) is applied to generate a 32-bit amplitude^2 Fourier power spectrum that is logarithmically scaled to enhance detection of high frequencies. Middle panel shows a diagrammatic representation of the window function that is multiplied to each raw data image before applying the FFT. This removes “edge artifacts” that otherwise appear as stripes along the axes of the Fourier spectrum of unprocessed data (bottom left, arrowheads). (**c**) An intermediate FFT stack is assembled (*Stack FFT* utility) and a maximum intensity projection generated over all three angles combined. For the final output the central region is masked before rescaling to enhance the contrast of the relevant higher frequency range. The processing steps enable at a glance to assess the presence and quality of first (inner) and less intense second order (outer) spots, corresponding to first and second order stripes in the structured illumination pattern.

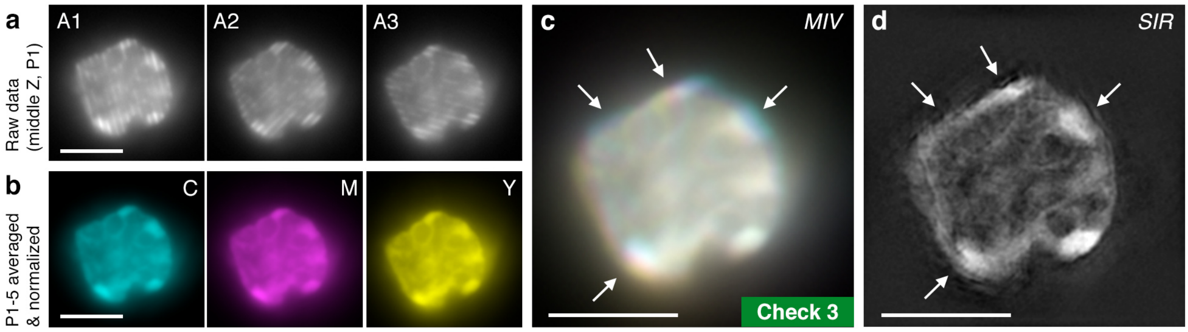

Supplementary Figure S5 | *Motion & Illumination Variation* check. (**a**) Raw data from a DAPI stained nucleus of a fixed, non-adherent mouse hematopoietic stem cell, showing one phase of each angle of a representative mid section (A1-A3 respectively). Data acquired on a GE OMX V2 instrument. (**b**) Data from **a** with average projection of all 5 phases, intensity normalization and color-coding (cyan, magenta, yellow) by angle. (**c**) Merge of the three panels in **b** result in a largely white "colocalization" image, but motion artifacts are illustrated around the edges of the nucleus, indicated by one colour predominating (arrows). (**d**) Reconstructed data from **a**, showing artifacts resulting from motion (arrows). Scale bar: 2 μm.

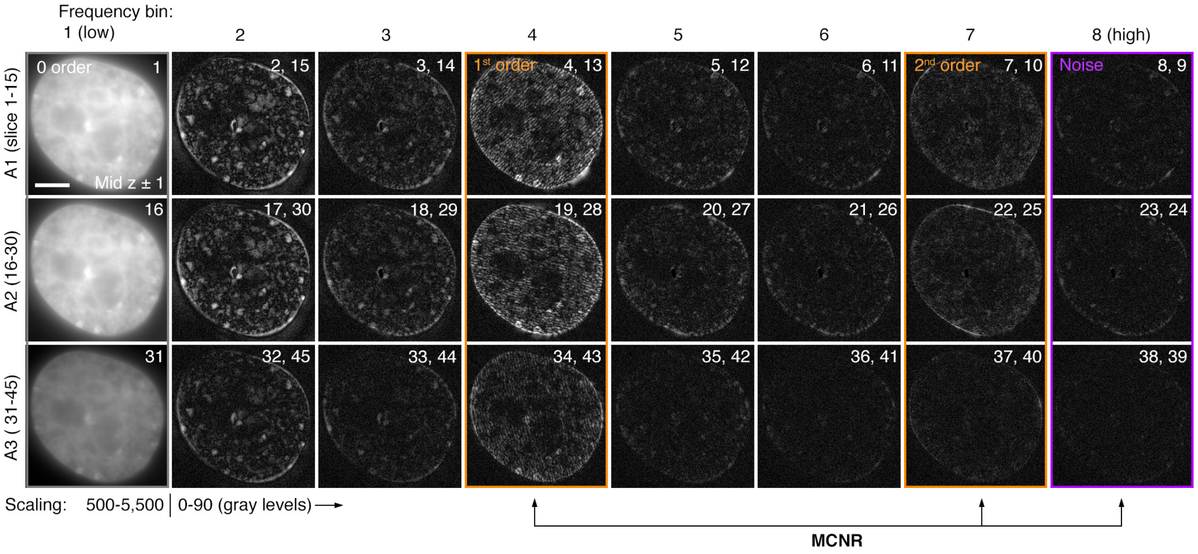

Supplementary Figure S6 | *Modulation contrast-to-noise* (MCN) calculation. Intermediate processing steps of the raw data corresponding to **Fig. 2e** is shown after 1D discrete Fourier transformation along the phase dimension to separate frequency components in the central ±1 z-planes (see **Methods**). Rows are arranged by angle (1-3), while slices indicate origin of each subsequent panel in each row. For the final MCN output all three angles are combined. Columns, containing equivalent data from each angle, indicate steps in processing, and progress from left to right in order of frequency information, from low to high. Numbers in the panels indicate their absolute position in the stack during processing, and ascend or descend relative to the position of the first (leftmost) column. The first, fourth, and seventh columns represent information generated by zero, first, and second-order stripes resulting from the interference of the structured illumination pattern and the sample, while the intermediate panels show intermediate steps taking during processing in *SIMcheck*. The rightmost column shows the noise contribution to the preceding images in each row. The MCNR value is calculated on a per-pixel basis as the ratio between and the first- and second order contribution shown in the fourth and seventh column (average of each pixel) and the noise contribution (1x StdDev over the whole image) shown in the rightmost column. Scale bar: 5 µm.

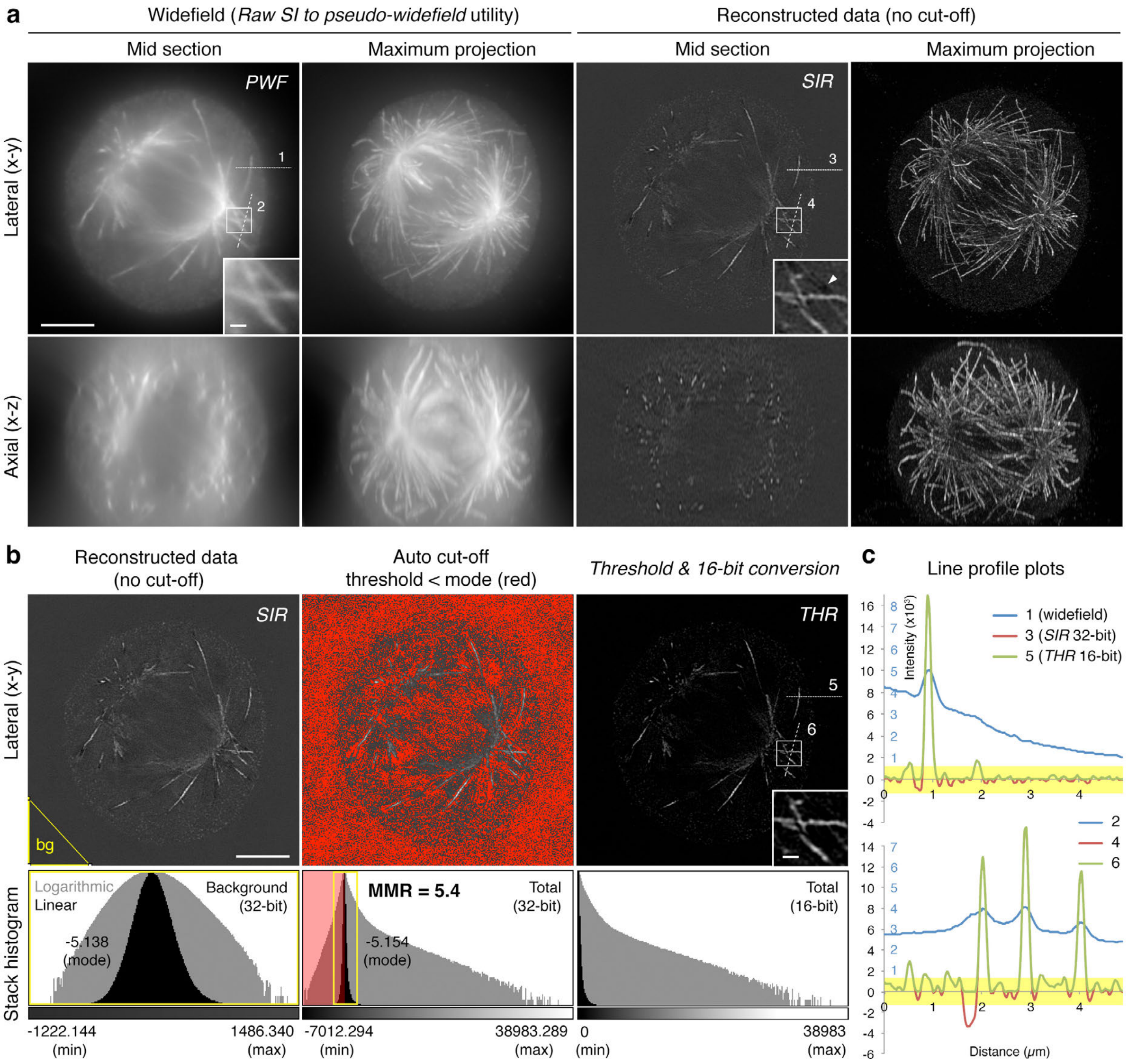

Supplementary Figure S7 | Image processing tools and intensity histogram analysis. (**a**) Dataset recorded of a mitotic HeLa cell immunostained for microtubules acquired on a GE OMX V2 instrument (see also **Supplementary Fig. S3**). Left panels: Conventional widefield resolution image stack was generated with the *Raw SI to Pseudo Widefield* utility. Right panels: Auto-scaled reconstructed data without further post-processing (**Supplementary Data 2b**). Grey colour in the background area of the mid section is due to the presence of darker regions above and below brighter features (inset, arrowheads). Maximum intensity projecting of the entire stack levels the background intensities due to the stochastic nature of the reconstruction noise. Scale bar: 5 µm (inset 0.5 µm). (**b**) Left panel: Stack histogram of the background area (bg, yellow triangle in lateral section) indicates a symmetric (Poisson) distribution of the noise around the modal value between ~ ±1,300 grey levels. Central panel: Pixel intensity values below the mode are coloured in red. The yellow box in the stack histogram indicates the range of the reconstruction noise. Values below this band originate from intensity dips surrounding brighter features. These become more prominent under suboptimal imaging conditions, particular with spherical aberration mismatch. The min-to-max-ratio (MMR) provides a metric for the degree of reconstruction artifacts in the dataset. Right panel: Same data set after applying the *Threshold & 16-bit Conversion* utility.

All values below the stack mode (default option, valid if the data contains a sufficient background area) are truncated and the dataset converted to a 16-bit composite TIFF without contrast stretching. (**c**) Intensity profile plots along the lines indicated in panels **a** and **b** demonstrating the x-y resolution doubling and massive contrast enhancement in the reconstructed data compared to the conventional widefield image. Reconstruction noise (yellow band) fluctuates around the mode threshold (~0; this value may vary between datasets depending on camera offset setting and sample conditions). After the noise cut-off, about half of pixel values within the background regions are still above this threshold, thus avoiding the uncontrolled eradication of reconstruction artifacts. Scale bar: 5 μm (inset 0.5 μm).

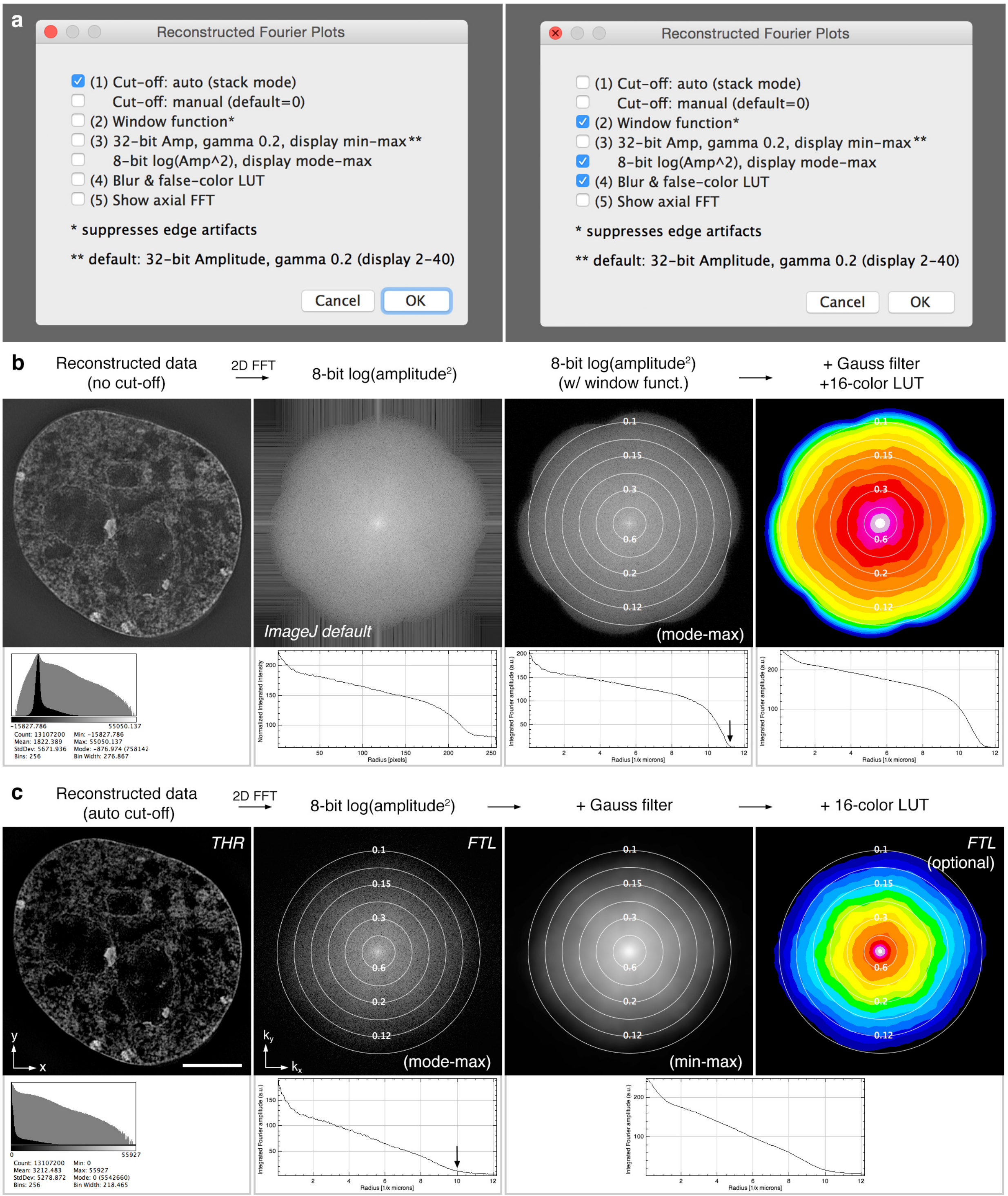

Supplementary Figure S8 | Display options for *Reconstructed Fourier Plot*. (**a**) Screenshot of the option menu specifically available to the stand-alone plugin, with default settings (left) and optional settings activated (right). (**b**) Display options without input data cut-off. Left panel: lateral mid section of the same reconstructed dataset as shown in **Fig. 3** with corresponding stack histogram and statistics displayed below. Second panel: 8-bit converted log-scaled (amplitude^2) power spectrum as generated by *ImageJ*'s default FFT function, with corresponding radial profile plot below. Third panel: *SIMcheck* result with active window function and display set to *8-bit log(amplitude^2)*, to remove edge artifacts and

amplify the high frequency range (compared to the default gamma correction; see **Fig. 3**). Overlaid concentric rings denote the respective spatial resolution (in µm). Arrow in the radial profile indicates the system- and wavelength-dependent limit of the frequency support (1/~11 µm = approx. 90 nm). Note that the resolution on the x-axis is provided as reciprocal distance. Right panel: display option with additional blurring and false-colouring to visually support the gradient characteristics of the frequency distribution by concentric coloured rings. (**c**) Left panel: Mid section of the same dataset after applying auto cut-off (threshold < stack mode). Second panel: log(amplitude^2) power spectrum and corresponding radial profile show less prominent drop-off due to removal of reconstructed noise in the negative range of the 32-bit reconstructed data. Arrow points to the inflection point between amplitude drop-off and FFT background that provides an approximation of the effective resolution limit in the data (1/~10 µm = approx. 100 nm). Intermediate blurring step before (third panel) and after false-colouring (right panel).

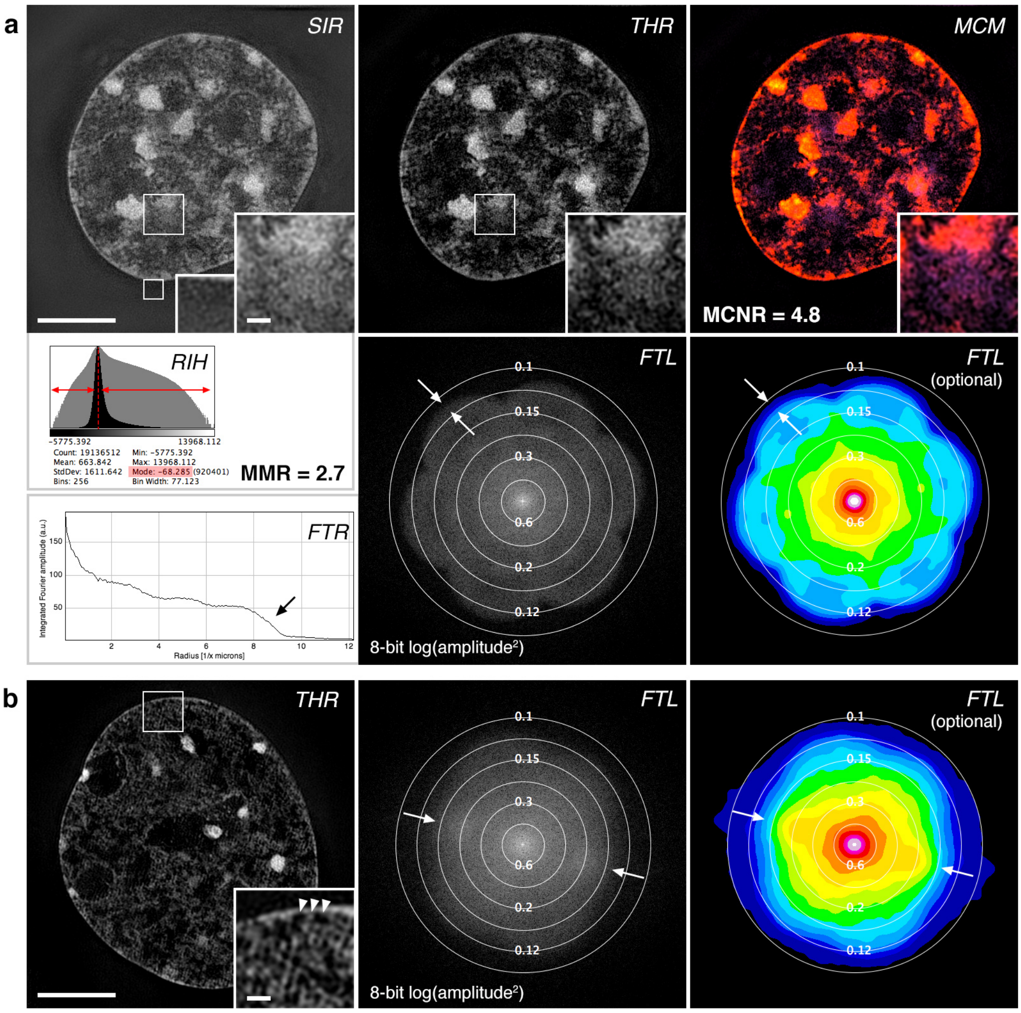

Supplementary Figure S9 | Identification of signal-to-noise and angle-specific artifacts. Reconstructed data from DAPI stained mouse C127 cell nuclei. (**a**) Mid section of unprocessed reconstructed data shows prominent small-scaled "curly" features (large inset, top left panel). Similar albeit weaker features are apparent from reconstructed noise in background regions (small inset, top left panel). These structures are still visible after clipping to the mode (inset, top middle panel). Mapping of the local MCNR values indicate very low underlying modulation contrast in the corresponding raw data (inset, top right panel) and a low average feature MCNR of 4.8 (threshold for acceptable reconstruction ~8). The corresponding intensity histogram provides a min-to-max ratio (MMR) of 2.7 (threshold of ~3 for acceptable reconstruction). The corresponding log-scaled Fourier plots (FTL, FTR) of the thresholded data show a sharp drop-off at the edge of the frequency support (arrows, lower panels). (**b**) Mid section of thresholded reconstructed data showing a stripy appearance in one angle direction (arrowheads in inset, left panel). The lateral Fourier plots show a skewing in the corresponding perpendicular orientation (middle and right panel, arrows), indicating reduced resolution in one angle, in this case caused by a false k0 angle reconstruction parameter.

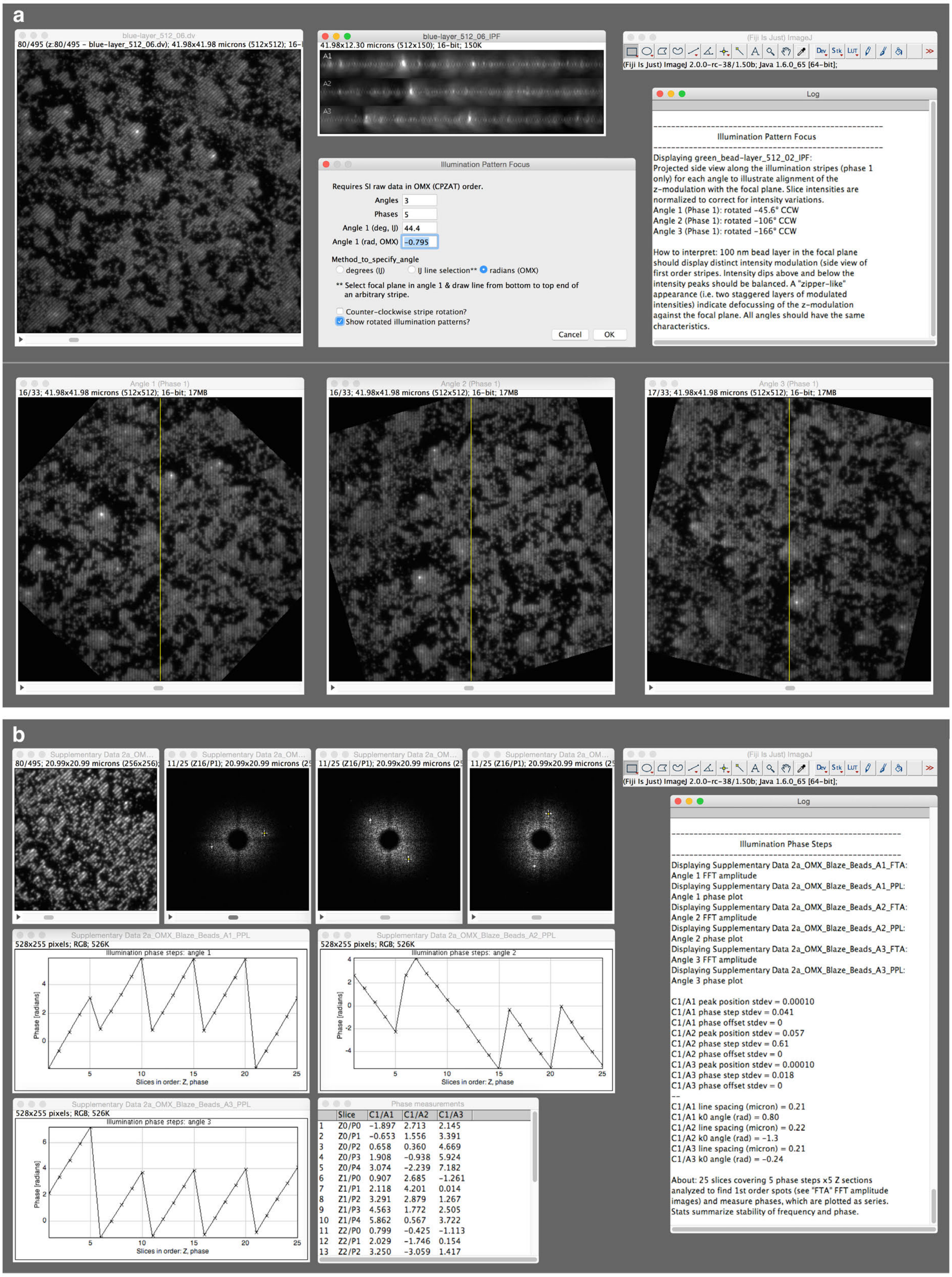

Supplementary Figure S10 | *Illumination Pattern Focus* and *Illumination Phase Steps* calibration tools. (**a**) Screenshot of the *Illumination Pattern Focus* tool applied on a raw SI dataset of a field of blue 110 nm fluorescent beads (FluoSpheres 350/440; Life Technologies) acquired with a GE OMX V3 Blaze system. Top left panel: Raw data

window. Top central upper panel: Result window with maximum intensity projected orthogonal views of the 1st order stripe pattern along the bead layer. Top central lower panel: Main dialog providing different options to define the first pattern rotation angle. Top right panel: Log output, including degree of rotation for each angle of the raw data, as well as suggestions for interpretation. Lower panel: Optional display of the separated angles (phase 1 only, images of phase steps 2-5 discarded) after rotation to the same vertical orientation. (**b**) Screenshot taken after running the *Illumination Phase Step* calibration on a dataset acquired from a green 100 nm bead layer with a GE OMX V3 Blaze instrument. Top left panel: Raw data window. Top central panels: angle specific (angles 1-3, respectively) FFT stacks for all phase positions within a defined z-range (in this example ±2 z-sections around plane of best focus). Central area with the highest amplitude is blocked. Yellow crosses indicate the pixel position of the highest intensity spots (identified by the *ImageJ*'s *Find Maxima* process), typically associated with the first order stripes of the illumination pattern. The windows below display plots of the phase values in radians at these spots for each angle (angles 1-3, respectively), as well as the measured values in a results table for export into a spreadsheet program for further analysis. The log window on the right provides information on the angle and stripe width.

Supplementary Table S11 | *SIMcheck* beta testers

Name	Affiliation	Location
Julio Mateos Langerak	CNRS Montpellier	FR
Theresa Swayne	Columbia University	US
Andreas Bruckbauer	Crick Institute London	UK
Edmundo Sanchez Guajardo	CSF Vienna Biocenter	AT
Lijuan Zhang	CSF Vienna Biocenter	AT
Kareem Elsayad	CSF Vienna Biocenter	AT
Andre Lampe	FMP Berlin	DE
Jan Schmoranzer	FMP Berlin	DE
Martin Lehmann	FMP Berlin	DE
Milos Sramek	GMI Vienna	AT
Matthew Pearson	IGMM University of Edinburgh	UK
Ann Wheeler	IGMM University of Edinburgh	UK
Jaron Liu	IMB Singapore	SG
Karin Aumayr	IMP Vienna	AT
Pawel Pasierbek	IMP Vienna	AT
Debora Keller	Imperial Collage London	UK
Tristan Piolot	Institut Curie Paris	FR
Orestis Faklaris	Institut Jacques Monod Paris	FR
Audrey Salles	Institut Pasteur Paris	FR
Eva Wegel	John Innes Centre Norwich	UK
Grant Calder	John Innis Centre	UK
Damir Sudar	Lawrence Berkeley National Laboratory	US
Felix Kraus	LMU Munich	DE
Katrin Schneider	LMU Munich	DE
Elke Küster-Schöck	McGill University	CA
Michael Cammer	New York University	US
Henrik Strahl von Schulten	Newcastle University	UK
Anil Shukla	NIH/NCI	US
Mariana Pinho	NOVA University of Lisbon	PT
Alison North	Rockefeller University	US
Monica Hasegan	Samuel Lunenfeld Research Institute	CA
Andrew Olson	Stanford University	US
John Mulholland	Stanford University	US
Manish Butte	Stanford University	US
Xiaoxue Zhou	Stanford University	US
Kathie Amberg-Johnson	UC Berkeley	US
Samantha Lewis	UC Davis	US
Stephen Ogg	University of Alberta	CA
Pascal Detampel	University of Calgary	CA
Alex Sossick	University of Cambridge	UK
Mark Bowen	University of Cambridge	UK
Nicola Lawrence	University of Cambridge	UK
Laure Plantard	University of Copenhagen	DK
Markus Posch	University of Dundee	UK
Peter Carlton	University of Kyoto	JP
Benjamin Hibbs	University of Melbourne	AU
Paul James McMillan	University of Melbourne	AU
Kay Oliver Schink	University of Oslo	NO
Richard Parton	University of Oxford	UK
George McNamara	University of Texas MD Anderson	US
Vito Mennella	University of Toronto	CA

SUPPLEMENTARY DATA

Download:
https://www.dropbox.com/sh/4deho8b9cyjb8u7/AABWRZvfGu6M2B6hnmz_qLxxa?dl=0

Supplementary Data 1 | OMX V3 Blaze example dataset 1. (**a**) Raw data and (**b**) reconstructed data (SIR) of a mouse C127 cell nucleus with chromatin stained with 4',6-diamidino-2-phenylindole (DAPI, blue, channel 1) and the proliferating cell nuclear antigen (PCNA) immuno-labelled with Alexa 488 (green, channel 2).

Supplementary Data 2 | OMX V2 example dataset. (**a**) Raw data and (**b**) reconstructed data (SIR) of a HeLa cell in metaphase with α-tubulin immuno-stained.

Supplementary Data 3 | OMX V3 Blaze example dataset 2. (**a**) Raw data and (**b**) reconstructed data (SIR) of a dense layer of green fluorescent 100 nm diameter beads (505/515 FluoSpheres, Life Technologies).

Supplementary Data 4 | Zeiss Elyra example dataset. (**a**) Raw data and (**b**) reconstructed data (SIR) of a three-color labelled human tissue culture cell nucleus (courtesy of S. Tashiro).

Supplementary Data 5 | Nikon N-SIM example dataset. (**a**) Raw data and (**b**) reconstructed data (SIR) of a three-color labelled human liver cell section (courtesy of C. O'Connell).
